# Recognizing Complex Upper Extremity Activities Using Body Worn Sensors

**DOI:** 10.1371/journal.pone.0118642

**Published:** 2015-03-03

**Authors:** Ryanne J. M. Lemmens, Yvonne J. M. Janssen-Potten, Annick A. A. Timmermans, Rob J. E. M. Smeets, Henk A. M. Seelen

**Affiliations:** 1 Research School CAPHRI, Department of Rehabilitation Medicine, Maastricht University, Maastricht, the Netherlands; 2 Adelante, Centre of Expertise in Rehabilitation and Audiology, Hoensbroek, the Netherlands; 3 BIOMED Biomedical Research Institute, Hasselt University, Diepenbeek, Belgium; 4 Department of Rehabilitation Medicine, Maastricht University Medical Centre, Maastricht, the Netherlands; University of Pennsylvania, UNITED STATES

## Abstract

To evaluate arm-hand therapies for neurological patients it is important to be able to assess actual arm-hand performance objectively. Because instruments that measure the actual quality and quantity of specific activities in daily life are lacking, a new measure needs to be developed. The aims of this study are to a) elucidate the techniques used to identify upper extremity activities, b) provide a proof-of-principle of this method using a set of activities tested in a healthy adult and in a stroke patient, and c) provide an example of the method’s applicability in daily life based on readings taken from a healthy adult. Multiple devices, each of which contains a tri-axial accelerometer, a tri-axial gyroscope and a tri-axial magnetometer were attached to the dominant hand, wrist, upper arm and chest of 30 healthy participants and one stroke patient, who all performed the tasks ‘drinking’, ‘eating’ and ‘brushing hair’ in a standardized environment. To establish proof-of-principle, a prolonged daily life recording of 1 participant was used to identify the task ‘drinking’. The activities were identified using multi-array signal feature extraction and pattern recognition algorithms and 2D-convolution. The activities ‘drinking’, ‘eating’ and ‘brushing hair’ were unambiguously recognized in a sequence of recordings of multiple standardized daily activities in a healthy participant and in a stroke patient. It was also possible to identify a specific activity in a daily life recording. The long term aim is to use this method to a) identify arm-hand activities that someone performs during daily life, b) determine the quantity of activity execution, i.e. amount of use, and c) determine the quality of arm-hand skill performance.

## Introduction

Patients with neurological diseases such as stroke or cerebral palsy often experience serious problems with arm-hand use in daily life [1,2]. Loss of arm-hand function—for example, due to a hemiparesis—limits the execution of activities of daily living (ADL) or arm-hand activities, resulting in greater dependency, restricted social participation and decreased quality of life [3,4].

Assessment of arm-hand skill performance is important for both clinical practice and research. In clinical practice it can, for example, be used to track the patient’s progress in arm-hand use during rehabilitation and information about arm-hand performance can be used to tailor treatment more effectively to the specific needs and goals of the individual patient. In research, assessment of arm-hand performance can be important to determine the effectiveness of (new) therapies or to determine the natural course of a disease regarding arm-hand problems. Many instruments exist to assess arm-hand use in several domains of the International Classification of Functioning, Disability and Health (ICF) [5]. For instance, measuring a patient’s arm-hand *function*, measuring his *capacity* to perform activities or gauging *(self-)perceived performance* [6]. However, instruments to assess *actual performance* in daily life are scarce.

The currently available instruments that measure actual performance both validly and reliably [6] are not capable of measuring a) which activities a patient actually performs with his arm and hand, b) the quantity of activity execution, i.e. amount of use, or c) the quality of arm-hand skill performance. Furthermore, they have several other major disadvantages. For instance, video observation [7] has the disadvantage that the recordings must be assessed by (multiple) experts, making the method potentially subjective and, moreover, highly time consuming. Furthermore, video observation intrudes severely on the patient’s privacy. Accelerometry, which is also used to measure actual arm-hand use [8], is unable to distinguish between specific activity-related movements and non-functional or unintentional movements, thus potentially overestimating a patient’s arm-hand use [9,10]. Furthermore, upper extremity accelerometry is rather insensitive for measuring changes over time, as was demonstrated in an intervention study by Lemmens et al. [9], which showed no improvement of actual arm-hand use as measured by accelerometry, even when patients reported improvements in arm-hand use at home for specific activities. A measure of actual performance that is able to identify and quantify specific activities might have shown improvements in this clinical intervention study. This illustrates the need for a more specific actual performance measure. In addition, accelerometry, as used currently, does not measure the quality of arm-hand performance [9,10]. The latter, however, is rated “very important” by both patients and therapists since it is associated with the ability to use the arm-hand while executing ADL.

Given these needs, a new actual performance measure that can not only identify specific arm-hand activities but also measure the quantity and, preferably, also the quality of specific arm-hand performance, should be developed. Such a measure needs to meet at least the following requirements if it is to detect actual arm-hand performance: 1) the measurement should be objective, i.e. not requiring subjective interpretations by the patient and/or the therapist, 2) the instrument should be portable and unobtrusively wearable for ambulatory use in daily life conditions, 3) the instrument should be able to identify specific activities and provide measures of arm-hand use as well as quality of arm-hand performance, and, finally 4) the instrument should be applicable in different patient populations [6].

The first step, and the focus of this paper, is to develop and elucidate a method for identifying specific arm-hand related activities. An instrument for assessing actual performance may be created with body-worn sensors that record movements in terms of specific movement patterns associated with specific activities. A combination of accelerometers, gyroscopes and magnetometers for measuring acceleration, angular velocity and orientation towards the earth’s magnetic field, is expected to be very useful for this purpose. To identify activities, pattern recognition techniques can be used. There are several pattern recognition approaches that can be used to identify activities. In general, pattern recognition can be divided into: a) supervised classification, where the input is classified into a predefined category, and b) unsupervised classification, where the input is classified into a so-far unknown category based on similarity of patterns [11]. Commonly used pattern recognition approaches are statistical classification, neural networks, structural matching and template matching [11,12]. The latter approach is used in the present paper, in which similarity between two entities—for example, signal profiles—will be determined. Signals from the accelerometers, gyroscopes and magnetometers may all show a specific pattern related to the movement executed, i.e. the performance of an activity. The combination of all these movement patterns (= multi-array signal patterns) may characterize specific activities and may be used as a template to recognize activities. The recording in which the activity must be recognized will be matched against the stored template and a correlation measure will be calculated.

The aim of this study was to a) elucidate the techniques used to identify upper extremity activities, b) provide a proof-of-principle for this method using a set of activities tested in a healthy adult and in a stroke patient, c) provide an example of the applicability in daily life based on a recording from a healthy adult.

## Methods

### Initial set of upper extremity activities

The present studythe technique for identifying activities elucidated using a set of three typical but distinctly different daily activities. These activities were chosen from a list of activities patients reported wishing to train and improve on [13,14]. In this study, the terms ‘activity’ and ‘task’ are equivalent, except that ‘activity’ refers to ADL in general while ‘task’ is used to refer to ADL executed after a verbal instruction. The activities ‘drinking from a cup’, ‘eating with knife and fork’, and ‘brushing hair’ were selected for two reasons. Firstly, this set of activities covered different levels of complexity [15], ranging from, for example, a kinematically simple activity like drinking to a kinematically far more complex activity like eating with a knife and fork. Secondly, it included two activities (drinking and brushing hair) with many similarities, the intention being to see whether it was possible to distinguish them even though they consisted of similar components, namely a reaching movement, grasping an object, bringing the object to the head and placing the object back on the table.

As in all upper extremity activities, each of these activities may be kinematically decomposed into multiple consecutive sub-phases or sub-tasks. A clinically used and ecologically valid, albeit descriptive, activity decomposition technique was described by Timmermans et al. [16]. However, as this technique was primarily based on the (implicit) expertise of clinical experts, an objective, computational technique for identifying sub-phases among upper extremity activities was used to detect specific activities among the daily activities that were recorded.

### Subjects

There are several aspects to this study. To provide proof-of-principle of the method for identifying activities in a standardized environment, thirty healthy subjects and one stroke patient (all aged over 50) participated in the study. To provide an example of the applicability of the method in daily life, an additional healthy adult was recruited for a 30-minute daily life recording. Healthy participants were recruited by advertisement among the staff of Adelante Rehabilitation Centre and their families. The one patient included in this study was identified in the Adelante Rehabilitation Centre database by a rehabilitation physician of the stroke unit and contacted by letter. The study was reviewed and approved by the Adelante Rehabilitation Centre’s Medical Ethics Committee in Hoensbroek, the Netherlands, and by the Medical Ethical Committee of Maastricht UMC+ (Maastricht, The Netherlands, NL42965.068.12). Before starting the measurements, written informed consent was obtained from all participants. The patient’s age, hand dominance and gender were recorded when they entered the study.

### Measurements

**Fig 1:**
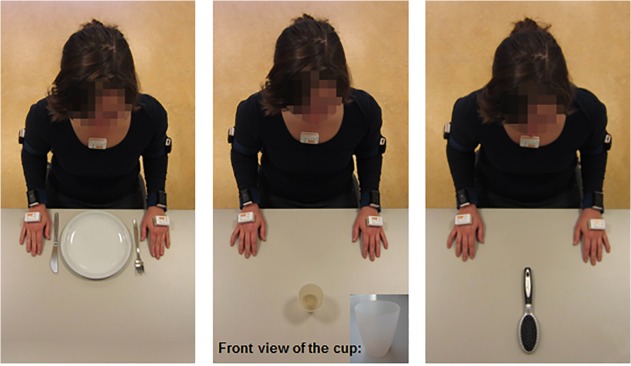
Overview of the positioning of the task utensils in the standardized setting.

Subjects performed the tasks ‘drinking from a cup’, ‘eating with knife and fork’, and ‘brushing hair’ in controlled conditions. They sat comfortably on a chair behind a table, both of which were height-adapted to the length of the participant to create a starting position in which their back was straight, their feet on the floor, their knees and hips flexed 90 degrees, their elbows flexed 90 degrees and the ulnar side of their hands touching the tabletop. For this study, all task utensils (empty cup, knife, fork, plate with play-dough ‘food’, and hairbrush) were arranged in a standard way on the tabletop (Fig. 1). After a brief verbal instruction about the task (Table 1), subjects performed each task five times at their self-selected speed, with short resting intervals in which they assumed the initial sitting position.

**Table 1:** Verbal instructions given to the participants.

**Drinking from a cup**	Reach towards the cup with the dominant hand, grasp the cup with one hand, bring the cup to your mouth, take a sip, return the cup to the original location, return to starting position.
**Eating with knife and fork**	Reach towards knife and fork, grasp knife and fork, prod with the fork in the food, use the knife to cut a piece off, bring the fork to your mouth, return knife and fork to the original location, return to starting position.
**Brushing hair**	Reach towards the hairbrush, grasp the hairbrush, bring the hairbrush towards the top of your head, brush the hair downwards twice on the side on which you are holding the hairbrush and twice on the other side, return the hairbrush to the original location, return to starting position.

In addition to the controlled conditions, a 30-minute daily life recording was performed in the home environment of the aforementioned additionally-recruited healthy person. The only instruction given was to act as normally as possible, performing some (self-chosen) activities of daily living, including the activity ‘drinking’ at least three times. The participant was observed by a researcher who was at the participant’s home during this recording and kept a record of the activities.

### Sensor-based instrument

**Fig 2:**
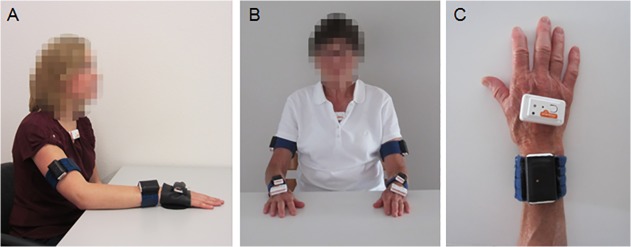
Placement of the sensor devices. A) placement on the dominant arm-hand and chest of healthy participants, B) bilateral placement on the arms, hands and chest of the stroke patient, C) top view of the placement of the sensor devices on the hand.

To unobtrusively measure the upper extremity kinematics during activity performance, four lightweight (27 gram, 52mm x 31 mm x 18 mm) wireless multi-sensor body-worn devices (9-DOF Shimmer, Shimmer Research, Dublin, Ireland), each consisting of a triaxial accelerometer, a triaxial gyroscope and a triaxial magnetometer, were used. One device was attached on the dorsal side of the hand (parallel to the *metacarpophalangeal joints*) using a custom-made glove, one device on the dorsal side of the wrist (directly proximal to the *proc*. *styloideus ulnea*) using a custom-made fabric strap with Velcro, one device on the upper arm (halfway between the *epicondylus lateralis* and the *tuberculum majus*, parallel to the longitudinal axis of the *humerus)* using a custom-made fabric strap with Velcro, and one device on the chest (located on the *manubrium sterni*) using hypoallergenic tape. The glove-like garment did not obstruct hand function. An overview of the device locations is shown in Fig. 2. The healthy participants performed the unimanual tasks with their dominant arm-hand. For the bimanual activity ‘eating’, the movements of the arm-hand manipulating the knife were recorded.

For taking readings from the stroke patients, the devices were attached not only to the dominant arm-hand and chest, but also to the non-dominant arm-hand. It was decided to measure both arms and hands because stroke patients use their affected arm-hand mostly as assisting arm-hand. It is important to measure the affected arm-hand because knowing the (quality of) movement of this arm-hand may be of use to the rehabilitation specialists and the patient, whereas the other arm-hand is mainly of interest because this arm-hand will make movements required to perform an activity (and is therefore necessary for the pattern recognition). For the unimanual tasks, the stroke patient used his unaffected arm-hand.

The wireless devices were connected to a computer via Bluetooth. Data were synchronously sampled and saved using the Multi-Shimmer Sync application, version 1.1.0 (Shimmer, Dublin, Ireland) at 256 Hz. For the measurement with the stroke patient, a sample frequency of 128 Hz was used to accommodate maximum throughput (number of devices * 9 channels * sample frequency * 2 bytes) of the recording system.

### Data pre-processing

All data processing was performed off-line using custom Matlab software (The Mathworks Inc., Natick, MA). Raw data consisted of 36 signals (four devices, each gauging three types of signals in three directions). Data from the stroke patient consisted of 63 signals (seven devices, each gauging three types of signals in three directions). To identify the dominant phases within the task performed, data pre-processing included zero-phase, low-pass filtering (4^th^ order Butterworth filter, cut-off frequency equal to 2.5 Hz) of all signals to obtain the low frequency signal content associated with the three-dimensional spatial displacements / kinematics of the task performed.

## Results

The results section is divided into three parts. The method for identifying activities will be elucidated (Part I) and proof-of-principle is shown using recordings under controlled conditions of healthy adults and a stroke patient (Part II) and a daily life recording in a healthy individual (Part III). This approach was used for all tasks described in the method section.

### Part I

Data analysis was aimed at:

- Identifying ‘templates’, i.e. combinations of multi-signal epochs representing a specific activity or sub-phase of a specific activity per subject, and
- Using these templates to uniquely identify performance of a specific activity among signals recorded during multiple regular daily activities.

This approach required several (consecutive) steps:

1. Temporal delimitation of each of the five attempts to perform a specific task, i.e. identifying the start and endpoint of each attempt recorded during the controlled task condition, based on the gyroscope signals.
2. Normalization of all signals (i.e. accelerometer, gyroscope and magnetometer) in the time domain of each attempt in order to correct for (small) variations due to differences in speed of task execution.
3. Creating templates by averaging all signal matrices (thus signals from all sensors from all devices) from the five attempts of each individual person to obtain the individual template (i.e. the underlying ensemble averaged signal matrix per task per individual), averaging signal matrices from the individual templates of multiple persons to create a generic template.
4. Identification of dominant sub-phases of templates (containing signals from all devices and sensors) within a specific task. The *time borders* of the sub-phases were identified using the gyroscope signals.
5. Recognition of specific activity execution among various activities performed daily, i.e. searching for template occurrence among signal recordings gathered in daily life conditions.

**Fig 3:**
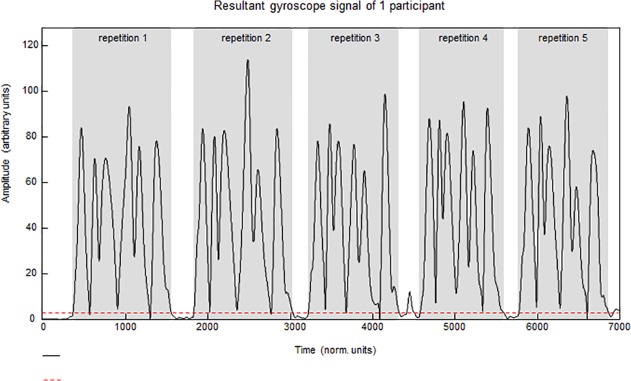
Procedure for identifying start and endpoints of task repetitions. The black line represents the summed gyroscope signal of four devices on one person (signal X). The dotted red line represents the threshold. Repetitions were identified using an algorithm making use of a threshold, minimal length of a repetition and minimum distance between two repetitions.

In Step 1 (temporal delimitation), the gyroscope signals were used to identify the time borders of the repetitions, i.e. the start and endpoints of each of the five attempts to perform a skill. A 3D resultant gyroscope signal was calculated for all Shimmer devices (Pythagoras’ rule). These 3D-resultant gyroscope signals were summed into one (artificial) resultant signal (X). This summation was performed to include the signal content of all gyroscope signals of *all* devices of one person, since it is not known beforehand which device (e.g. chest, upper arm, wrist or hand) will be moved first at the start of each task execution and which device will be moved last at the end of each task execution. From the resultant signal, X, a threshold was determined from the signal variance recorded during rest periods, thereby distinguishing the ‘resting’ signal from the ‘active’ signal. When the use of the standard threshold resulted in finding time points not associated with the performance of the task, in an iterative process, the threshold was increased until this problem was resolved. A minimum duration of the specific task at hand was set a-priori, as was a minimum duration of the rest phase between repetitions of the task (a-priori parameters, APPs), both expressed in data points, thereby avoiding false-positive findings regarding the tasks’ start and endpoints. A collection of five signal matrix epochs, each containing *all signals from all devices and all sensors* and representing one task execution, was obtained by using the start and endpoints identified. These signal matrix epochs were of unequal length since no instructions about performance speed were given. A schematic overview of the procedure for identifying start and endpoints for each attempt is shown in Fig. 3. When it was not possible to identify all repetitions using the described APPs, the APPS were adjusted manually and repetitions were identified using the same algorithm. Unsuccessful repetitions, caused by problems with the data transfer or incorrect execution of the activities (i.e. not following the instruction), were discarded.

In Step 2 (normalization of the signals in the time domain), each of the aforementioned five signal matrices (each containing 36 signals, except for the data from the stroke patient, which consisting of 63 signals) were linearly interpolated to a length of 1000 data points. The ensemble was then averaged to produce one time-normalized individual template signal matrix per task (Step 3) and a generic template was created. In the present study, the generic template was made by averaging the time-normalized individual template signal matrices from 29 of the 30 healthy persons for the measurements in the standardized environment. The template was then applied to the signals of the 30^th^ healthy person.

**Fig 4:**
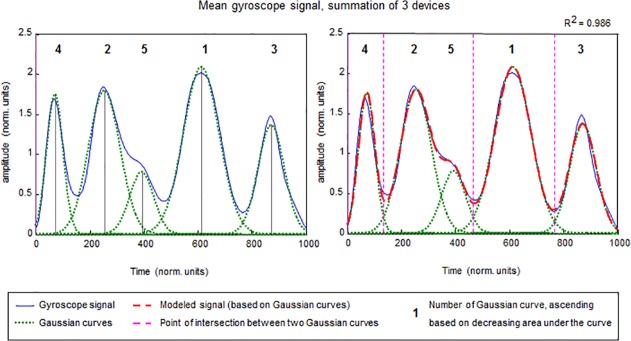
Identification of sub-phases of a task, based on modeling the summed gyroscope signal with non-normalized Gaussian curves. R^2^ displays the amount of signal variance explained.

Step 4 involved identifying dominant sub phases of templates. To identify the time borders of sub-phases, the 3D resultant gyroscope signal (as calculated in Step 1) from the time-normalized template of the devices on the hand, wrist and upper arm were summed to produce one overall resultant signal representing overall gyroscope signal content. This was done for both the individual template and the generic template. In order to identify dominant phases of upper extremity tasks, the summed gyroscope signal was modeled as a summation of non-normalized Gaussian curves of various lengths in a linear envelope decomposition procedure described by Chen et al. [17]. An example of this procedure is shown in Fig. 4.

Cross-correlating the modeled signal (based on Gaussian curves) to the original signal produced a “goodness-of-fit” measure (R^2^ or amount of variance explained). The number of Gaussian curves needed to optimally represent the original signal, was established in an iterative process guided by the trade-off between R^2^-improvement on the one hand and the amount of overlap between consecutive Gaussians (i.e. the uniqueness of a movement phase relative to its predecessor) on the other. The Gaussian curves were numbered according to their signal power (i.e. area under the curve), representing sub phase dominance within the total task. Next, the time points at which one Gaussian curve crossed its successor were computed (i.e. point of intersection between two Gaussian curves). These time points represent the time borders of ensuing sub-phases within a task, which can then be used to further identify sub-phase templates. In some cases, two Gaussian curves were combined to outline a sub phase. Such a combination was made if one of the Gaussian curves was too small or had too much overlap with another Gaussian curve. For instance, in Fig. 4 the Gaussian curves numbered 2 and 5 were combined. Using the time borders already identified, signal matrix epochs, each containing *all signals from all devices and all sensors* and representing sub phases of a task, were obtained.

In summary, at this stage in the overall analysis procedure, templates representing the complete task (Temp_com_) as well as templates for sub phases of the tasks (Temp_sub_) and their corresponding timing parameters have been identified, and will be used in Step 5, i.e. for recognizing specific activity execution among various daily activities. Templates consist of all signals from all devices and all sensors, thus 36 for the recordings with the healthy participants and 63 for the recordings with the stroke patient.

**Fig 5:**
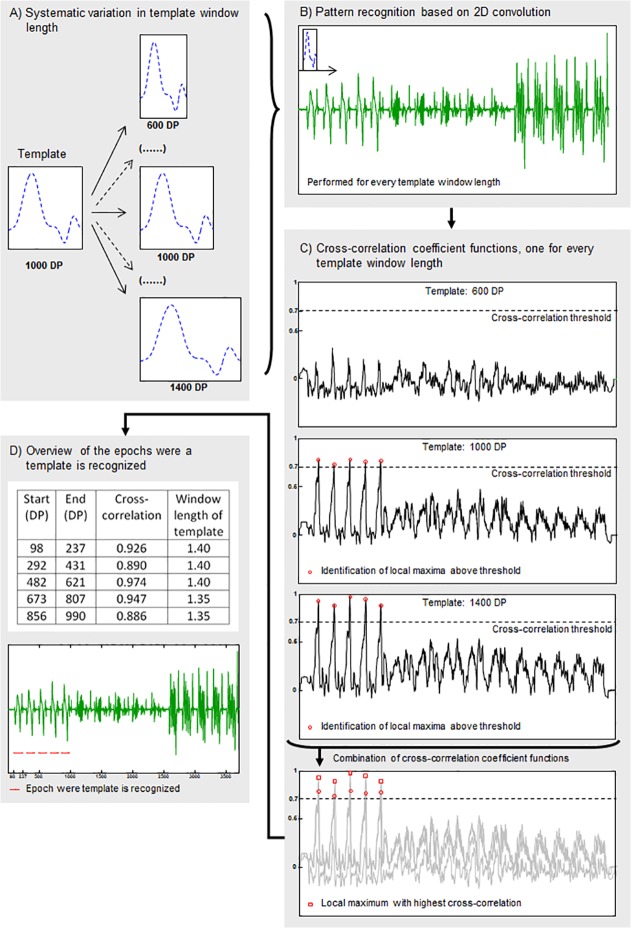
Schematic overview of the procedure of pattern recognition (1 of the 36 signals is shown). A) Systematic variation in template window length using linear interpolation, example given of 3 of the in total 17 variations in window length, B) Pattern recognition based on 2D convolution, performed for every template window separately, C) Cross-correlation coefficient functions, calculated during pattern recognition, example given of 3 of the in total 17 cross-correlation coefficient functions. Local maxima were identified per cross-correlation coefficient function (marked with red circle) and local maxima with highest cross-correlation of all cross-correlation functions were identified (marked with red square), D) Overview of epochs where the template is recognized, including start and endpoints (in data points, DP), cross-correlation coefficients and relative window length. Red lines mark the places in the sequence of recordings of multiple standardized daily activities were the template is recognized. DP = data point.

In Step 5, a pattern recognition algorithm based on 2D convolution was used to identify template occurrence among signal recordings made during daily activity performance. A schematic overview of the procedures described in Step 5 is shown in Fig. 5. The pattern recognition algorithm is used on all 36 signals simultaneously (or in the case of the stroke patient, in all 63 signals simultaneously). In the present study, as described earlier, the measurements were taken under standardized conditions, except for one measurement performed in the home situation of a participant. Because task duration in daily life is variable, template window length may not fully match actual task duration in daily life conditions. Consequently, an iterative procedure was used in which the template window length was varied systematically between 0.6 and 1.4 times its original size in increments of 0.05 (resizing factor), using linear interpolation (Fig. 5A). During each iteration, the 2D convolution procedure (Fig. 5B) created a normalized cross-correlation coefficient function representing the level to which the (resized) template matched a corresponding part of the daily activity signals recorded. All cases in which the normalized cross-correlation coefficient function exceeded a predefined threshold (minimal “goodness-of-fit” level) were identified. This pre-defined threshold was set manually. The threshold was task dependent and differed between the complete template and template sub phases. Next, local maxima of this resultant time series were calculated after each resizing iteration (Fig. 5C). Finally, each local maximum found per iteration was compared to its temporal equivalent from the other iterations, thus producing a) an array of correlation coefficients associated with ‘best fits’, b) an array of sizing factors associated with the aforementioned correlation coefficients, and c) two arrays of start and endpoints indicating the time frame in the daily activity recording in which the resized template was positively identified (Fig. 5D). Step 5 was performed for Temp_com_ and Temp_sub_ and for both the individual and generic templates.

The concept of recognizing specific activity execution among the various activities performed will be shown using typical examples of measurement data from the participants. If an activity was performed and recognized (i.e. using Temp_com_ or all sub phases of Temp_sub_ consecutively in time), it was classed as a true-positive finding. If an activity was not performed and not recognized, it was called a true-negative finding. If it was performed but not recognized it was called a false-negative finding. And if an activity was not performed but recognized with the pattern recognition algorithm, it was called a false-positive finding. Percentages of true-positive findings were calculated, i.e. (number of true-positive findings/number of times a task was performed)*100%.

### Part II

#### Demographics

To provide proof-of principle of the method for identifying activities in a standardized environment, thirty healthy participants and one stroke patient were included in this study for the measurements under standardized conditions. The healthy participants included 14 women and 16 men. Mean age was 58.0 ± 5.1 years. For all these participants the dominant arm-hand was the right arm-hand. The stroke patient was male and aged 57 (impaired arm-hand: left, dominant arm-hand: right) with moderate spasticity.

#### Recognising activities under standardized test conditions in a healthy individual

**Fig 6:**
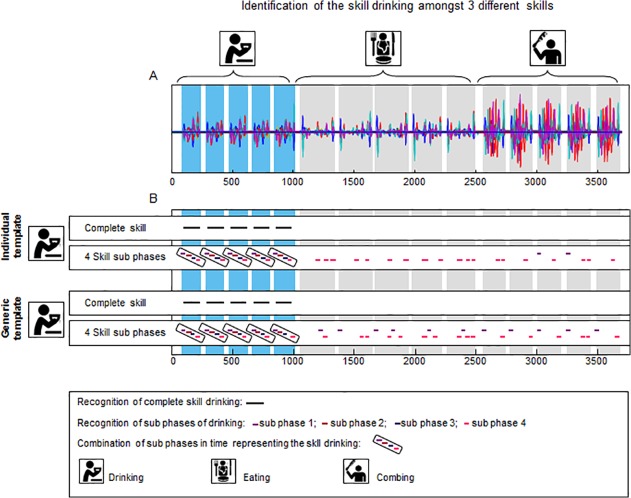
Identification of the activity ‘drinking’ in a sequence of recordings of multiple standardized daily activities of a healthy participant. Panel A displays the superimposed signals (36 in total) of a recording of an individual while executing three tasks, namely drinking from a cup, eating with knife and fork and brushing hair. Panel B displays the pattern recognition using an individual template and the pattern recognition using a generic template consisting of the mean signal of 30 persons. Both pattern recognition with the complete task as template and task sub-phases as template are shown. The black lines (complete task) and coloured lines (task sub-phases) in Panel B mark the places were the template is recognised in the longer recording.

Fig. 6 shows the identification of the task ‘drinking’ (= template) in a recording containing five repetitions of the task ‘drinking’, five repetitions of the task ‘eating’ and five repetitions of the task ‘brushing hair’ (= sequence of recordings of multiple standardized daily activities). Panel A shows the 36 signals of the sequence of recordings of multiple standardized daily activities. Panel B shows the recognition of the task ‘drinking’ using an individual template, and the recognition of the task ‘drinking’ using the generic template. When the individual template was used, drinking was identified with a high sensitivity and high specificity for both Temp_com_ and Temp_sub_. Five out of five drinking repetitions were recognized (100% true-positive). The mean correlation of this pattern recognition was 0.925 for Temp_com_ and ranged between 0.885 and 0.990 for Temp_sub4_. There were no false-positive findings. Using the generic template, the skill ‘drinking’ was also identified with high sensitivity and high specificity. The identification of the task ‘drinking’ was still 100% true-positive (identified five out of five repetitions). Although there were some false-positive findings some individual sub-phases (mean correlation ranging between 0.660 and 0.873), there were no false-positive findings for the complete tasks and the combination of all sub-phases. The mean correlations were 0.786 for Temp_com_ and ranged between 0.778 and 0.864 for Temp_sub_. In addition, there were no false-negative findings for either Temp_com_ or Temp_sub_ and for neither the individual nor the general templates.

**Fig 7:**
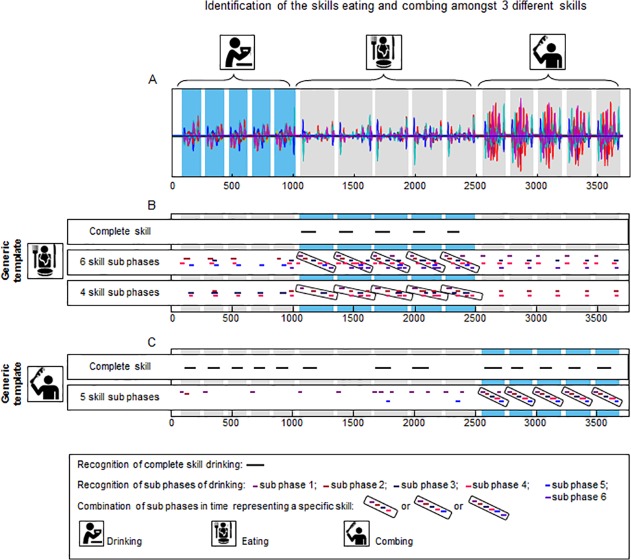
Identification of the activities ‘eating’ and ‘brushing hair’ in a sequence of recordings of multiple standardized daily activities of a healthy participant. Panel A displays the superimposed signals (36 in total) of a recording of an individual while executing three tasks, namely drinking from a cup, eating with knife and fork and brushing hair. Panel B shows the pattern recognition using a generic template for the task ‘eating’. Panel C presents pattern recognition using a generic template of the task ‘brushing hair’. Both pattern recognition using the complete task as a template and pattern recognition using task sub-phases as a template are shown. The black lines (complete skill) and coloured lines (skill sub-phases) in panels B and C mark the places were the template is recognised in the longer recording.

Fig. 7 shows the identification of the tasks ‘eating’ and ‘brushing hair’ in the sequence of recordings of multiple standardized daily activities that used the generic templates. As the outcome of the pattern recognition using the individual templates was equal to or even better than the outcomes using the generic templates for both the eating task and the brushing task, only the results for the generic template are shown. For the task ‘eating’, two templates consisting of sub-phases were constructed that differed in the number of sub-phases. One template consisted of six sub-phases (Temp_sub6_) as identified with the Gaussian curves described in the method section. For the second template (Temp_sub4_), two consecutive sub-phases were combined to form one longer sub-phase. This was done twice (sub-phases 1 and 2 were combined, as were sub-phases 4 and 5). Panel B shows that the task ‘eating’ is recognized with high sensitivity—five out of five repetitions were recognized (100% true-positive) using Temp_com_, Temp_sub6_ and Temp_sub4_. The mean correlation for this pattern recognition was 0.579 for Temp_com_, and ranged between 0.712 and 0.829 for Temp_sub6_ and between 0.693 and 0.800 for Temp_sub4_. False-positive findings occurred for some individual sub-phases (mean correlation ranging between 0.673 and 0.833), but no false-positive findings were present for the complete tasks and the combination of all sub-phases. There were also no false-negative findings for all templates. This indicates a high specificity for task recognition. Panel C shows the identification of the task ‘brushing hair’. The ‘brushing hair’ task is recognized in five out of five repetitions (100% true-positive) using both Temp_com_ and Temp_sub_. Mean correlation for the pattern recognition was 0.415 for Temp_com_ and ranged between 0.675 and 0.898 for Temp_sub4_. Using Temp_com_, there were eight false-positive findings (mean correlation of 0.422; five times drinking, three times eating) out of ten task performances of the activities ‘drinking’ and ‘eating’. Thus sensitivity was lower than for the tasks ‘drinking’ and ‘eating’. However, using Temp_sub_, false-positive findings occurred for some individual sub-phases (mean correlation ranging between 0.658 and 0.890), but there were no false-positive findings for the combination of all sub-phases. There were also no false-negative findings for either Temp_com_ or Temp_sub_, indicating high sensitivity.

#### Recognition of activities under standardized conditions in a stroke patient

**Fig 8:**
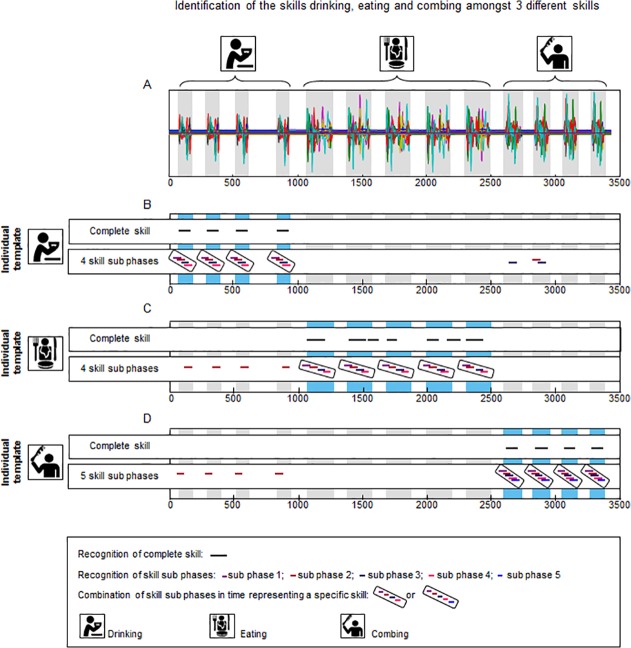
Identification of the activities ‘drinking’, ‘eating’ and ‘brushing hair’ in a sequence of recordings of multiple standardized daily activities of a stroke patient. Panel A displays the superimposed signals (63 in total) of a recording of a stroke patient while performing three tasks, namely drinking from a cup, eating with knife and fork and brushing hair. Pattern recognition using an individual template is shown for the tasks ‘drinking’ (Panel B), ‘eating’ (Panel C) and ‘brushing hair’ (Panel D). Pattern recognition with the complete task as template and with the task sub-phases as template are both shown. The black lines (complete task) and coloured lines (task sub-phases) in panels B and C mark the places were the template is recognised in the longer recording.

Fig. 8 shows the identification of the tasks ‘drinking, ‘eating’ and ‘brushing hair’ in the data of the stroke patient. The task ‘eating’ was performed five times and the tasks ‘drinking’ and ‘brushing hair’ were performed four times. Panels B, C and D show the pattern recognition with the individual template. For the pattern recognition with Temp_com_ all executions of the tasks ‘drinking’ (four out of four repetitions), ‘eating’ (five out of five repetitions) and ‘brushing hair’ (four out of four repetitions) were identified (100% true-positive) with no false-negative or false-positive identifications. Mean correlation was 0.99, 0.65 and 0.97 respectively for the tasks ‘drinking’, ‘eating’ and ‘brushing hair’. Pattern recognition with Temp_sub_ identified all tasks unambiguously (100% true-positive) with no false-negative or false-positive identifications. The mean correlation ranged between 0.81 and 0.99 for the task ‘drinking’, between 0.81 and 0.98 for the task ‘eating’, and between 0.60 and 0.99 for the task ‘brushing hair’. The tasks drinking, eating and brushing were therefore recognized with high sensitivity and high specificity. Since only one stroke patient was included in this study, no generic template could be created.

### Part III

#### Demographics

For the recording during daily life, one additional healthy subject participated (male, aged 32, dominant arm-hand: right).

#### Recognition of activities in a daily life recording

**Fig 9:**
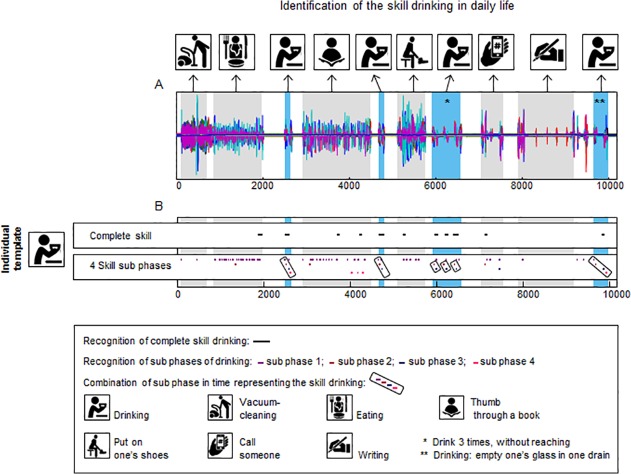
Identification of the activity ‘drinking’ in a recording in the home situation of a healthy participant, including multiple activities of daily living. Panel A shows the superimposed signals (36 in total) of the recording made at home. Panel B displays the pattern recognition using an individual template for the activity ‘drinking’. Both pattern recognition with the complete activity as template and with activity sub-phases as template are shown. The black lines (complete activity) and coloured lines (activity sub-phases) in Panel B mark the locations were the template is recognised in the longer recording.

Fig. 9 shows the identification of the activity ‘drinking’ in a daily life recording. This recording included the activities ‘vacuum-cleaning’, ‘eating with a spoon’, ‘thumbing through a book’, ‘putting shoes on’, ‘phoning someone’, ‘writing’, and ‘drinking’ (the latter performed six times, as marked blue in Fig. 9, with 3 drinking performances in the part of the recording marked with *). Since no instructions were given to the participant, the activity ‘drinking’ was performed differently (confirmed during observation of the participant) compared to the performance of drinking during the recording of the template under standardized conditions. The differences were: the initial position and the end position of the participant in all performances in daily life, the position of the cup, being lower and more to the left in the first and second drinking performances in Fig. 9, the absence of the reaching and grasping phase during the drinking performance marked with * in Fig. 9 (the cup was held with the hand in the lap), finally the ‘sipping phase’ in the drinking performance marked with ** in Fig. 9 took longer as it involved emptying the whole glass instead of taking one sip.

Using Temp_com,_ six out of six drinking repetitions were recognized (100% true-positive, indicating high sensitivity, mean correlation was 0.51) but there were also five false-positive findings (mean correlation was 0.46). Using Temp_sub4_, the first drinking performance was identified (correlation ranging between 0.70 and 0.82), and for the second drinking performance, three out of four sub-phases were identified (true-positive, correlation ranging between 0.62 and 0.74). For the drinking performance marked with *, sub-phases 1 and 4 were not identified (false-negative). False-positive findings were frequent for sub-phase 1 and sporadic for the other sub-phases (mean correlation between 0.55 and 0.92). However, there were no false-positive findings for the combined sub-phases.

## Discussion

### Aim and main findings

The aim of this study was to a) elucidate the techniques used to identify upper extremity activities, b) provide a proof-of-principle for this method using a set of activities tested in a healthy adult and in a stroke patient, c) to provide an example of the applicability in daily life using a recording from a healthy adult. This paper elucidated the method for identifying an activity and showed that it is possible to identify the activities ‘drinking, ‘eating’ and ‘brushing hair’ in a sequence of recordings of multiple standardized daily activities in a healthy individual by using templates based on individual signals, as well as by using generic templates. Furthermore, it was possible to identify the activities ‘drinking’, ‘eating’ and ‘brushing hair’ in a sequence of recordings of multiple standardized daily activities in a stroke patient (using an individual template). When the pattern recognition in a healthy individual was compared with that of a stroke patient, it could be seen that the stroke patient had no false-positive results. This may be because the movement pattern of patients is more characteristic for a specific activity. That is, the template signal profiles in patients differ more distinctly between different activities, whereas in healthy subjects sub-phase templates of different activities may be more similar.

The activities differed in complexity. Naylor and Briggs defined complexity of activities as the number of parts or components and the extent of information processing demands that characterize an activity [15]. More complex activities have more component parts and require more information processing than less complex activities [15]. The activity ‘drinking’ is less complex than eating, for several reasons. One of these is that eating is a bimanual activity, demanding more cognitive effort and bimanual coordination, and this is known to be more difficult than unimanual arm-hand use [15]. In the present study, less complex activities such as drinking and a more complex activity such as eating with knife and fork were both perfectly identified. Furthermore, activities which contain similar elements (task similarity) [15,18] were distinguished from each other. These included drinking and brushing hair, both of which involve reaching for and grasping an object as well as displacement of the object towards the head and back to the table. In addition, it was possible to identify the activity ‘drinking’ in a 30 minute daily life recording.

### Individual template versus generic template

The signals used for the template may originate from a recording from one person (an individual template) or from mean signals of multiple persons (a generic template). One advantage of a generic template is that it may be used for pattern recognition in many individuals. In this way, it is not necessary to create a set of individual templates representing different activities for each individual. Although the performance of upper extremity activities by patients using their impaired arm-hand will differ from the performance of healthy individuals, it is possible to construct a generic template consisting of signals from patient data. Nevertheless, it can be expected that the variability between patients will be too large to use a generic template. A solution to this problem may be to use an individual template for each patient. The extra measurement required to create this individual template can be done at the patient’s home just before the daily life measurement, instead of in the laboratory, which will reduce the burden on the patient. The method for identifying activities described in this study was illustrated with data from a healthy person and data from a stroke patient. With the healthy individuals, recognition of the activities ‘drinking’, ‘eating’, and ‘brushing hair’ could be performed with an individual template as well as with a generic template. For the identification of activities performed by the stroke patient, only the individual template was used (as currently no generic template based on data from multiple stroke patients is available). All three activities performed by the stroke patient were identified correctly.

### Template of the complete activity versus activity sub phases

A template may consist of signals representing the performance of a complete activity, or of signals representing sub-phases of the performance of an activity. In the present study different activities were identified unambiguously in a standardized environment, with the template representing the complete activity and the array of templates representing the activity sub-phases. However, the performance of activities in daily life will most certainly differ from the performance of the activity as measured when creating the template. For instance, the performance of the activity ‘drinking’ can vary according to the person’s initial posture (as seen in the daily life recording). This influences the reaching movement (= variance in pattern) and thereby affects the pattern recognition. Or there might be variability in the duration of the performance (= temporal shift), e.g. whether the person takes one sip from a glass or drinks it all at once. Similarly, the performance of the activity ‘eating’ may vary, for instance, according to the use of the knife and the number of cuts needed to cut the food. As performances of activities in daily life situations may vary considerably, both the variance in the signal pattern and the temporal shift between a) the template and b) the daily life recording will probably be more of a problem when using the template representing the complete task than with the template representing the sub-phases. As to the pattern recognition using sub-phases, if one specific sub-phase is not recognized adequately, the recognition of the other sub-phases may still lead to the whole activity being identified correctly. If in the performance of the activity ‘eating’, the template consists of cutting the food once with the knife, the sub-phase containing ‘cutting’ might be found multiple times in a row during a daily life recording in which the food is cut multiple times. Another advantage of using sub-phases is that additional non-task-related movements, such as head scratching, will not influence the pattern recognition because this additional movement will most likely occur between two sub-phases. The principle of breaking activities into sub-phases is similar to the idea of Bai et al, who suggested that movements can be understood in terms of smaller components called ‘movelets’ [19]. Bai et al. used movelets to predict activity types such as walking, standing and sitting.

### Laboratory-based versus daily life recording

The ultimate goal is to develop a method/measure to assess actual performance in daily life, i.e. measuring a) which activities a patient actually performs with his arm and hand, b) the amount of arm-hand use, and c) the quality of arm-hand skill performance. Results from the measurements taken in the standardized environment have shown that the current method is able to identify specific activities among a variety of activities. However, the translation from measurements in a laboratory situation to a daily life situation is difficult. Many instruments with promising results in a standardized environment turned out to be less efficient or not applicable in a daily life situation [20–22]. This is why the present study included a proof-of-principle test condition involving a daily life situation. Although the method described needs to be further optimized, the results presented in this paper show that it is possible to identify a specific activity (drinking in this case) in a daily life situation. However, it should be noted that the recording shown in the present paper only lasted 30 minutes. Future research should include longer daily life recordings. The next section describes how the method should be optimized for identifying activities in daily life.

### Optimising the pattern recognition method

The identification of activities is based on resemblance between the signal patterns of the template and the signal patterns of the daily activity recording. This resemblance is expressed as a cross-correlation coefficient function. Currently, the cross-correlation threshold above which local maxima will be identified is set manually. In future, the level of the threshold should be set automatically within the pattern recognition algorithm itself.

Sub-phases were identified based on decomposition using Gaussian curves that identified dominant phases within the gyroscope signals recorded per task. With this method, it is possible to identify very short sub-phases. However, sub-phases of shorter duration result in shorter sub-phase template lengths with less specific signal contents. This makes it more likely that two different sub-phases will be identified as similar, leading to false-positive findings. For the task ‘eating’ it was shown that the template consisting of 6 sub-phases was capable of identifying the activity, but with many false-positive findings. After combining 2 sub-phases into 1 larger sub-phase (twice), a template consisting of 4 sub-phases was created that led to fewer false-positives but was still able to identify ‘eating’. In the future, the eligibility of combining sub-phases should be automated as part of the pattern recognition algorithm.

In general, the computational time needed for the data-analysis is in the order of seconds. However, the time currently needed for procedures in which some settings have to be made manually mostly falls between 1 and 3 minutes, after which computational time, again, was in the order of seconds. Consequently, the time needed for data analysis could be shortened by optimizing manual procedures.

For the pattern recognition, a total of 36 signals (for the healthy participant) or 63 signals (for the stroke patient) were used. That is, the x, y and z axes were evaluated separately for each device and each sensor. In this way, the placement and orientation of the sensor can affect the output on each axis. At the moment, this seems to have no large effects on the pattern recognition. However, when it becomes a problem—for instance, in a daily life situation—the calculation of a 3D resultant signal for each sensor in every device might be a solution. This, however, needs to be investigated further.

For identifying activities in a daily life situation, a combination of the template containing the complete activity performance and the array of templates containing activity sub-phases will probably be best.

### Optimizing the actual performance measure

For the activity performance by the healthy individuals, sensors were attached to the dominant arm and hand of the participants. The dominant arm-hand plays a major role in performing both unimanual and bimanual tasks. For the healthy subjects, recordings from sensors on the dominant arm-hand were successfully used to identify specific activities. In patients with impairments of their arm-hand, however, there is also clinical interest in measuring the affected arm-hand. Unimanual activities will be most likely performed with the unimpaired arm-hand, but the affected arm-hand plays an important role in performing bimanual activities. Therefore, for the measurement with the stroke patient, bimanual recordings were made. That is, measuring both the impaired arm-hand and the non-impaired arm hand. It was shown that identification of both unimanual and bimanual activities was achieved using the data from sensors attached to the trunk and to both arms and hands. In future, signals of the impaired arm-hand will additionally be used to assess, for instance, the quality of performance during specific activities.

The current paper has described the principle and practice of identifying activities using ambulatory sensors containing a tri-axial accelerometer, tri-axial gyroscope and tri-axial magnetometer. To be applicable in daily life situations, however, the sensor system needs to be adapted to be even less obtrusive and more user-friendly. For example, the sensors should be smaller so they can be worn under the clothes or incorporated in the fabric of clothes [23]. Furthermore, the data should be saved locally or streamed to a device or mobile phone that the participant can wear, instead of streaming it to a computer.

### Comparison with actual performance measures described in the literature

The ultimate aim is to develop an actual performance measure capable of a) identifying which activities a patient actually performs with his arm and hand, b) measuring the quantity of executing activities (i.e. the amount of use), and c) assessing the quality of arm-hand skill performance. As far as we know, no such instruments are currently available. However, many instruments are being developed to assess actual performance. Many different pattern recognition techniques are available and are being used. Leutheuser et al, for example, used a feature set of four time domain features and two frequency domain features and a combination of classification systems to distinguish between activities such as vacuuming, sweeping, sitting, standing, cycling, ascending/descending stairs and walking [24]. Their proposed classification method had a mean positive classification rate of 89.6%. With the meta-classifier AdaBoost M1 they achieved an overall accuracy of 95% for recognizing a set of activities that included walking, lying, making a sandwich, cleaning windows and computer work. Healthy participants wore five wireless accelerometers and performed the activities in standardized as well as in semi-naturalistic circumstances. Classification using subject-independent data (leave-one-out method) outperformed classification with user-specific data [24]. Some instruments use static or non-body-worn sensors in addition to ambulatory sensors such as the SERSMAA instrument [10] or the Data Acquisition Platform (DAP) described in Min and co-workers [25]. The SERSMAA is capable of detecting the handling of specific tagged objects, while the DAP is able to classify early morning activities. A disadvantage of systems that use static sensors is that they are harder to implement because the sensors must be installed in a patient’s home or because only specific objects can be used. Strohrmann et al used 10 ETH orientation sensors (consisting of 3D accelerometers, 3D gyroscopes and 3D digital compasses) to evaluate quality of movement during the execution of motor tasks [26]. They assessed, amongst others, task completion time, movement intensity, smoothness of movement and synchrony of arm movements.

The system and method used in the present paper differed to some extent from the studies mentioned above. For example, we focused solely on upper extremity activities. From a clinical point of view, problems with arm-hand skill performance are very important and require a thorough assessment instrument. In order to be able to recognize multiple upper extremity activities and distinguish between these activities, it is essential to measure multiple body segments of the upper limb. Furthermore, we divided the recorded signals into several consecutive sub-phases. Apart from improving pattern recognition, this approach may also be very useful in quantifying the quality of arm-hand performance, as these sub-phases can be seen as the building blocks of the total activity. However, it must be noted that other pattern recognition approaches exist, each having their own advantages, and it is likely that no one single pattern recognition approach is optimal. Jain et al stated that multiple methods and approaches have to be used, i.e. combining several sensing modalities and classifiers [11]. The pattern recognition approaches and techniques that have additional benefits to the present method need to be explored. More research is needed to further improve the actual performance measures.

### Future research

Future research should focus on optimizing the method for identifying activities, quantifying actual arm-hand use and measuring the quality of actual arm-hand performance. Furthermore, the concept as described in the present paper should be applied to more activities and in more (patient) populations. As the instruments should be applicable in both adults and children, the concept should also be tested in children as their motor control and motor planning differs from that of adults [27,28], resulting in different movement patterns (signal patterns of activities to be recognized). Thereafter, this concept should be extensively tested in the daily life situations of individuals.

### Conclusion

In the present study, we have elucidated a technique for identifying activities and presented a proof-of-principle with data from a healthy individual and from a stroke patient. It can be concluded that this method is very promising with regard to the applicability of these sensor devices for identifying specific activities among multiple activities, both among healthy individuals and patients, and in both a standardized setting and in daily life conditions.
